# Functional anatomy and ion regulatory mechanisms of the antennal gland in a semi-terrestrial crab, *Ocypode stimpsoni*

**DOI:** 10.1242/bio.20147336

**Published:** 2014-05-02

**Authors:** Jyuan-Ru Tsai, Hui-Chen Lin

**Affiliations:** 1 Department of Life Science, Tunghai University, Taichung 40704, Taiwan; 2 Center for Tropical Ecology and Biodiversity, Tunghai University, Taichung 40704, Taiwan

**Keywords:** Antennal gland, Osmoregulation, Na^+^, K^+^-ATPase, Labyrinthine cell, Coelomic cell

## Abstract

Brachyuran crabs from diverse habitats show great differences in their osmoregulatory processes, especially in terms of the structural and physiological characteristics of the osmoregulatory organs. In crustaceans, the antennal glands are known to be important in osmoregulation, and they play a functional role analogous to that of the vertebrate kidney. Nevertheless, the detailed structure and function of the antennal glands in different species have rarely been described. The aim of this study is to investigate the role of the antennal gland in ion regulation by examining the ultrastructure of the cells and the distribution of the ion regulatory proteins in each cell type in the antennal gland of a semi-terrestrial crab. The results showed that Na^+^, K^+^-ATPase activity significantly increased in the antennal gland after a 4-day acclimation in dilute seawater and returned to its original (day 0) level after 7 days. Three major types of cells were identified in the antennal gland, including coelomic cells (COEs), labyrinthine cells (LBRs) and end-labyrinthine cells (ELBRs). The proximal tubular region (PT) and distal tubular region (DT) of the antennal gland consist of LBRs and COEs, whereas the end tubular region (ET) consists of all three types of cells, with fewer COEs and more ELBRs. We found a non-uniform distribution of NKA immunoreactivity, with increasing intensity from the proximal to the distal regions of the antennal gland. We summarise our study with a proposed model for the urine reprocessing pathway and the role of each cell type or segment of the antennal gland.

## INTRODUCTION

Osmoregulation is an important process in aquatic crustaceans. Osmoregulators maintain their haemolymph or urine osmolality during environmental stresses. The overall osmoregulatory process involves absorbing or excreting ions between the environmental medium and the body fluid through osmoregulatory organs, such as gills, antennal glands and the gut in decapod crustaceans (Chung and Lin, 2006; Freire et al., 2008). The antennal gland is an excretory organ in crustaceans, although its functions differ among species (Holliday and Miller, 1984; Freire et al., 2008). The antennal gland is involved in the anion transporting process to modulate the loss of ions due to urine excretion (Holliday and Miller, 1984; De Vries et al., 1994; Péqueux, 1995; Morris, 2001; Freire et al., 2008). In marine decapods, the antennal gland plays a major role in nitrogen excretion and a minor role in contributing to ionic regulation (Holliday and Miller, 1984; Péqueux, 1995; Freire et al., 2008). In the euryhaline mud crab *Scylla paramamorsain*, Na^+^, K^+^-ATPase (NKA) activity in the antennal glands increased slightly after crabs were transferred to diluted seawater for 12 hr (Chung and Lin, 2006).

In aquatic crabs, gills are the main site for ion absorption; however, in terrestrial and land crabs, ion recruitment from the environmental medium is restricted by the loss of gill function in the presence of a dehydrating stress (Péqueux, 1995; Morris and Adamczewska, 1996; Morris et al., 1996). A land-living decapod must retain ions and water by reducing the loss of urine. The conservation of ions from the urine in aquatic crustaceans is an important issue in freshwater adaptation. Functional shifts of ion regulatory ability between the gills and the antennal gland have also been observed. For example, isosmotic urine was found to be produced by the antennal gland without ion reabsorption in *Carcinus maenas* (Riegel and Lockwood, 1961) and *Uca pugnax* (Green et al., 1959). In certain other crustaceans, such as the crayfish *Procambarus blandingi* (Peterson and Loizzi, 1973; Peterson and Loizzi, 1974), the Dungeness crab *Cancer magister* (Wheatly, 1985) and the semi-terrestrial *Ocypode quadrata* (De Vries et al., 1994), the antennal glands can reabsorb ions and produce hyposmotic urine. In the land-living terrestrial crab *Gecarcoidea natalis*, isosmotic urine was produced and redirected into the branchial chamber for ion reabsorption (Morris and Ahern, 2003). The roles of the antennal glands in the ion regulatory process vary among crustaceans.

The antennal gland of brachyuran crabs can be viewed as a structural analogue of the mammalian nephron (Wheatly, 1985; De Vries et al., 1994; Brown et al., 2009). The antennal gland consists of a complex labyrinth and coelomosac, and the labyrinth contains ion regulatory cells similar to those in the proximal tubule of the mammalian kidney (Freire et al., 2008). In the ghost crab *Ocypode quadrata*, in which the ion composition of the urine and haemolymph differs, the antennal gland has been hypothesised to reprocess the anion content (De Vries et al., 1994). However, De Vries and colleagues did not present the detailed structure of the antennal gland. The structure–function relationship of the antennal gland in osmoregulation in crustaceans has seldom been examined (Schmidt-Nielsen et al., 1968; Holliday and Miller, 1984; Freire et al., 2008). A more detailed description of the antennal gland has only been presented for the fiddler crab *Uca mordax* (Schmidt-Nielsen et al., 1968). Those authors suggest that the antennal gland contains at least two cell types, the coelomic cell (COE) and the labyrinthine cell (LBR). However, the details of the functional differentiation of the different parts of the antennal gland in brachyuran crabs remain unclear.

In this study, we furnish a through description of the structure of the antennal gland and propose potential roles for the antennal gland in the ion regulatory mechanism in a semi-terrestrial crab, *Ocypode stimpsoni*. We (1) investigated the ability of the antennal gland for ion regulation, (2) clarified the cell components and the ultrastructure of different portions of the antennal gland, (3) localised several ion-regulatory proteins in each type of cell and (4) proposed a refined ion transport model for the antennal gland.

## RESULTS

### The Na^+^, K^+^-ATPase (NKA) activity of the antennal gland

**Fig. 1.**
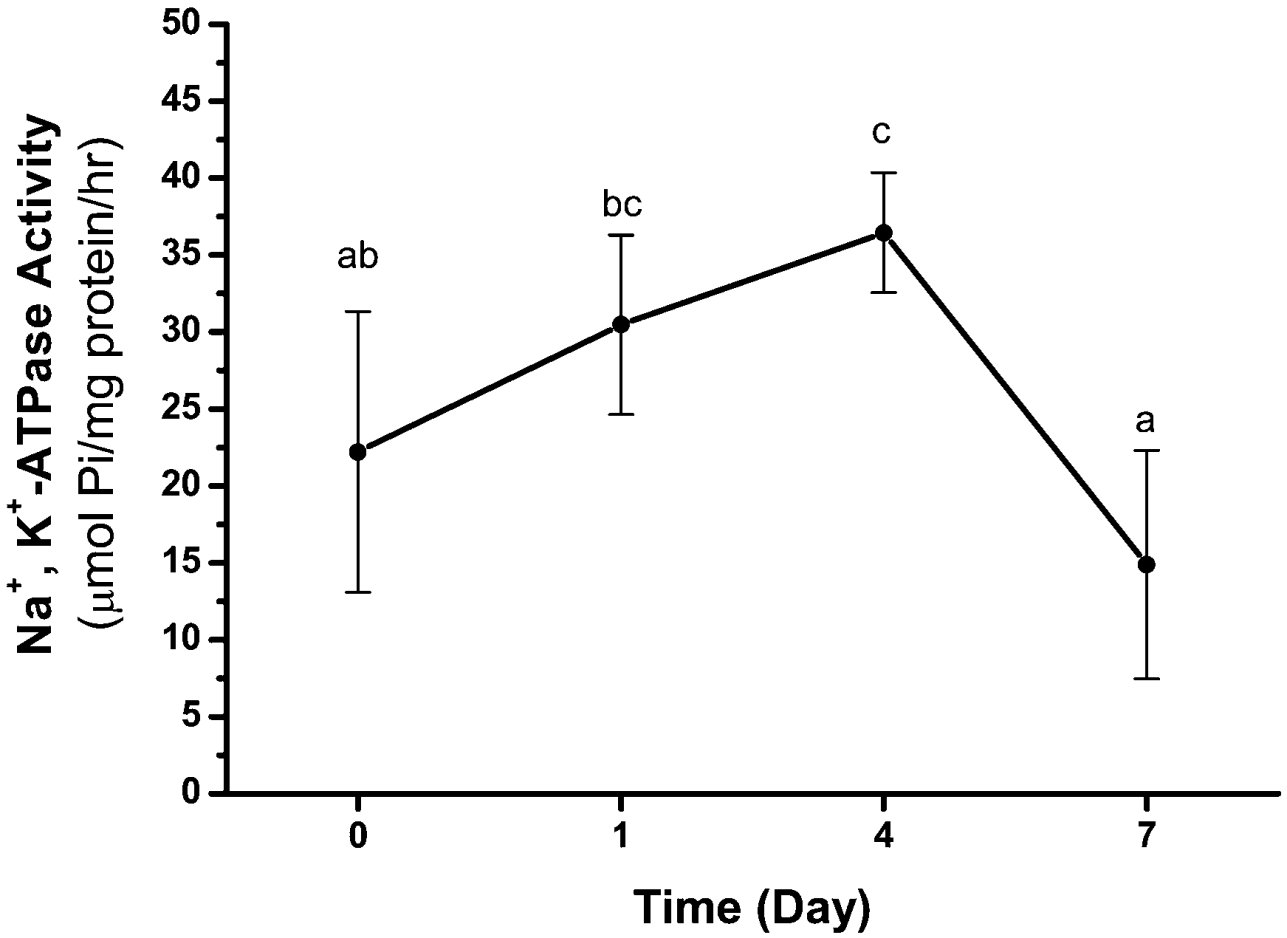
The Na^+^, K^+^-ATPase (NKA) activity in the antennal gland of *Ocypode stimpsoni* in 0, 1, 4 and 7 days after crabs were transferred to 5‰ seawater. Compared to the control group (0 day), the NKA activity increased and reached a significant level at the 4th day followed by a return to the normal level at the 7th day. These results are analyzed by one-way ANOVA (*p*<0.05) and grouping by Duncan's multiple range test. Different letters indicate significant differences (*p*<0.05).

When the crabs were transferred from 35‰ to 5‰ seawater, the antennal gland NKA activity increased on the 1st day after transfer to 5‰ diluted seawater and attained a significant difference on the 4th day (F_(3,25)_ = 10.17, *p*<0.01, *n* = 5–11), then decreased to the control (35‰) level on the 7th day (Fig. 1).

### The gross anatomy of the antennal gland

**Fig. 2.**
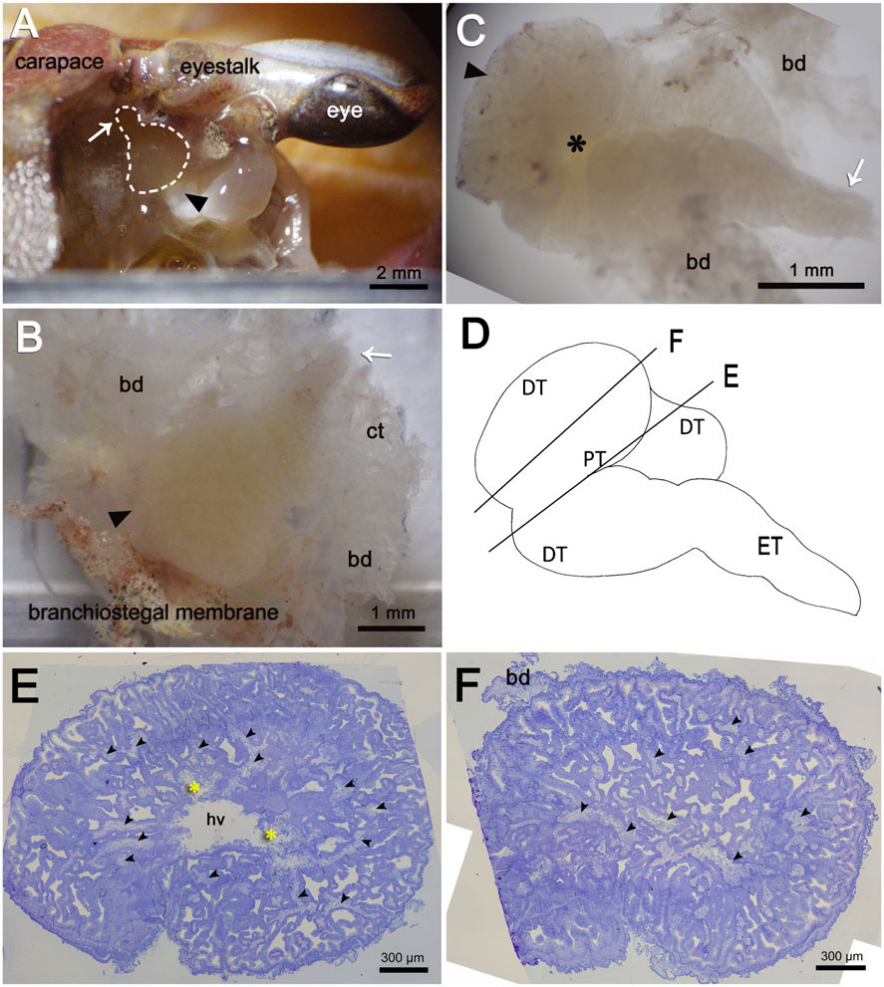
The gross anatomy and the portions of the antennal gland. (A) The antennal gland is located in the base of the eyestalk (as the dash circle shows). The arrowhead (◂) indicates the anterior portion of the antennal gland, and the arrow (←) indicates the posterior portion. (The dorsal view of the crab with the carapace removed. The upper side of this figure is the front of the crab.) (B,C) The removed antennal gland is coiled with the bladder (bd) and connective tissue (ct). (B) The dorsal view of the antennal gland has a smooth surface. (C) The ventral view of the antennal gland has an indentation (*) in the centre of the anterior portion. (D) The anterior portion of the antennal gland is composed of the proximal tubular region (PT) and the distal tubular region (DT). The posterior portion of the antennal gland is composed of the end tubular region (ET). Lines E and F represent the different sectioning levels of the antennal gland. In section (E), the sectioning level is crossing through the anterior part and includes both PT and DT. Tubular cells and large coelomosac (arrowheads) are found in the peripheral of the antennal gland defined as DT. A large haemolymph vessel (hv) is found in the centre of the antennal gland which is defined as PT. The haemocytes (*) are found in the haemolymph vessel (hv). In section (F), the sectioning level is on the peripheral region of the antennal gland and includes only DT. Numerous tubular cells and large coelomosac (arrowheads) filled up this region. Scale bars: 2 mm (A), 1 mm (B,C), 300 µm (E,F).

The antennal gland is located in the base of the eyestalk in the suborbital region (Fig. 2A). The antennal gland is 3–5 mm in length and 2–3 mm in width in an average individual with a carapace width of 35±5 mm covered by connective tissue and the bladder sac (Fig. 2B,C), the antennal gland is extremely soft and fragile. The fresh tissue is yellowish to pale in colour. According to orientation and structure, the antennal gland can be divided into two portions: the anterior portion and the posterior portion (Fig. 2D). The anterior portion is round and smooth on the dorsal side (Fig. 2B) and bears an indentation on the ventral side (Fig. 2C). The proximal tubular region (PT) and the distal tubular region (DT) are located in the anterior portion; the end tubular region (ET) is located in the posterior portion along with the linings of the connective tissues and the bladder (Fig. 2B–D). In the PT, a large central haemolymph vessel occurs and is the entrance for the coelomosac artery (Fig. 2E). This large vessel structure is not found in the peripheral region (DT) of the anterior portion of the antennal gland (Fig. 2F). In summary, the antennal gland is composed of anterior and posterior portions, which are further divided into the PT, the DT and the ET (Fig. 2D).

### The cell types and structures of the antennal gland

**Fig. 3.**
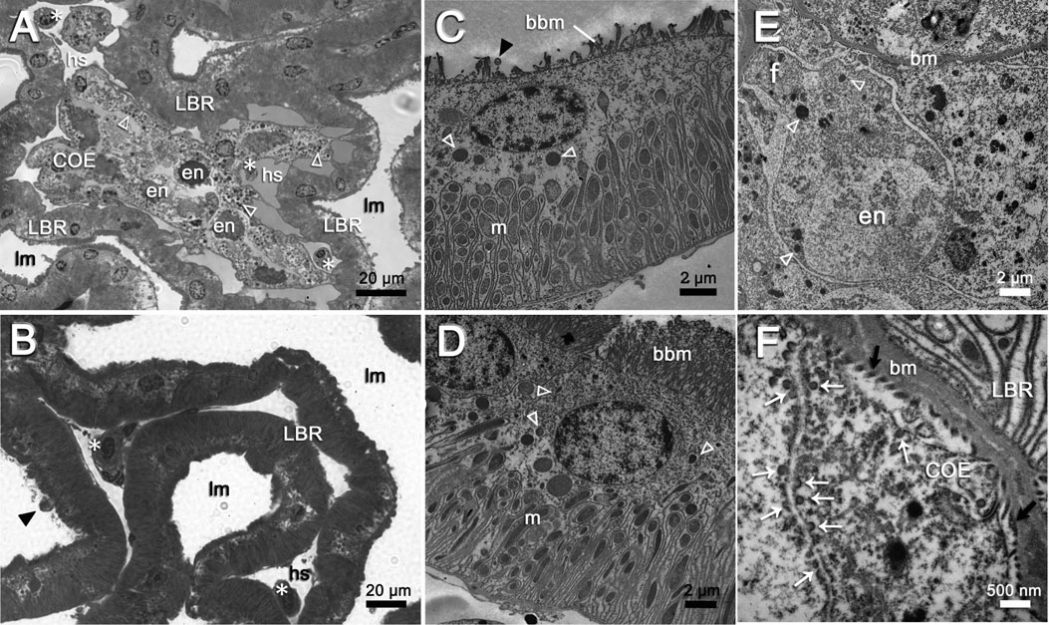
The cell arrangement and the two major cell types, COEs and LBRs, in PT and DT of the antennal gland. (A,B) Haemolymph vessels (hv) are composed of endothelial cells (arrowheads) in between COEs and LBRs. The endothelial lining of the haemolymph vessel (hv) is connected to the COEs directly (arrow). Haemocytes (*) are only found in the haemolymph sinus (hs) or in the haemolymph vessels (hv). Lumen (lm) is always in the apical side of LBR. No haemocyte was found in the lumen (lm). (C,D) LBRs are cuboidal cells with apical brush-boarder microvilli (bbm), basolateral membrane folding and lots of mitochondria (m). Numerous vesicles (open triangles) are found in the sub-apical spaces. The length of the apical brush-boarder microvilli (bbm) of LBR increases from that in PT (C) to that in DT (D). (E) COE has irregular cell shape and has extended foot process (f) attached to the basal matrix (bm). Numerous vesicles (open triangle) are found in the COEs. Some of the COE contain a large endosome (en), or called vacuole, in the cytoplasm. (F) The enlarged picture of the foot process shows pedicels (black arrows) along the basal matrix (bm) and has very small vesicles (white arrows), or called the pinocytes, in the cell margin of COE. Scale bars: 20 µm (A,B), 2 µm (C–E), 500 nm (F).

**Fig. 4.**
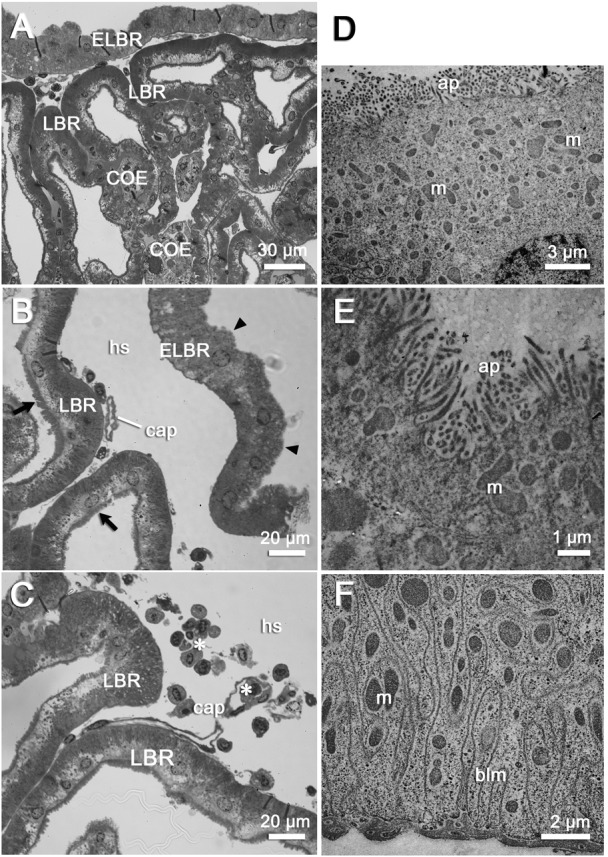
The ultrastructure of the labyrinthine cells (LBRs) and end-labyrinthine cells (ELBRs) in the end tubular region (ET) of the antennal gland. (A) ET is the end portion of the antennal gland and is composed of LBR, ELBR and COE. (B) ELBRs are always found in the peripheral side of ET. ELBRs have loose apical microvilli (arrowheads) while LBRs have the brush-boarder microvilli (black arrows). Capillaries (cap) are found between the basal side of the LBRs and ELBRs. (C) Haemocytes (*) are found in the capillary and the haemolymph space (hs). (D) The upper side of the ELBR shows irregular apical surface without a distinct sub-apical region. The mitochondria (m) are distributed from the base of the apical membrane to the top of the nucleus (located in the bottom-right of this photo). (E) The mitochondria (m) are distributed just below the apical surface. (F) The ultrastructure of the bottom side of ELBR. The mitochondria (m) are distributed in the whole cytoplasm and also twined with the basal membrane folding (blm). Scale bars: 30 µm (A), 20 µm (B,C), 3 µm (D), 1 µm (E), 2 µm (F).

The continuous simple cuboidal labyrinthine cells (LBRs) are the main cell type of the antennal gland. These LBRs interdigitate with the round coelomic cells (COEs) from the PT to the DT (Fig. 3A,B), and the LBRs are replaced by another cell type, the end-labyrinthine cell (ELBR), at the very end of the ET (Fig. 4). The entrance of the coelomosac artery is in the PT region, and the artery branches into several large haemolymph spaces in the PT and the DT (Fig. 2E). Connections between the endothelial cells of the haemolymph vessels and the COE are often found in the PT (Fig. 2E, Fig. 3B). Haemocytes are only found in the haemolymph vessels and in the haemolymph sinus located between the endothelial cells and the basal membrane of the COEs (Fig. 3A,B); they never appear in the lumen of the antennal gland. A well-developed endosomal system is found in the COE and is categorised into the pinocyte (50–150 nm), the vesicle (200–750 nm) and the vacuole (>1 µm, a large endosome) according to the diameter of the structure (Fig. 3E,F). Generally, a large vacuole is found on the top (near the lumen) of the COE (Fig. 3E). Pinocytes are usually generated from (or excreted to) the membranes of the extended foot process and the canal between two COEs (Fig. 3F).The LBRs have a well-developed brush-border membrane on the apical side and membrane folding and mitochondria on the basal side (Fig. 3C,D). The total thickness of the cell and the length of the apical microvilli increase from the PT and the DT to the ET, but the ratio of the length of the apical microvilli to the total thickness of the cell remains the same (Fig. 3C,D; Table 1). Vesicles are distributed in the apical and sub-apical regions of the LBRs, but no mitochondria occur in the sub-apical region (Fig. 3C,D). The COE has an irregular shape, with an extended foot process attached to the basal matrix (Fig. 3E,F).

**Table 1.**
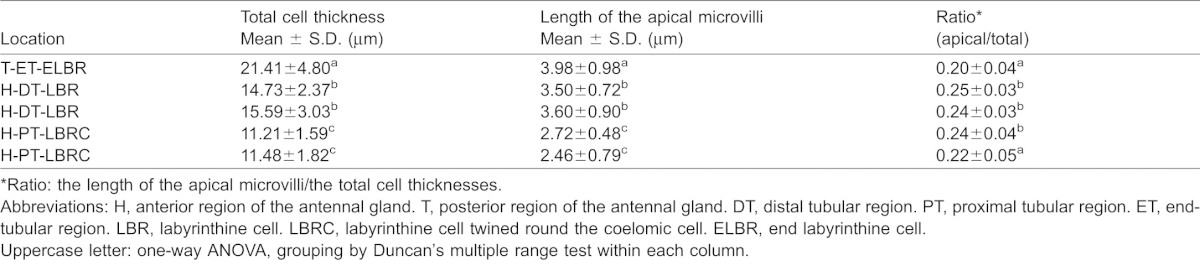
The cell thickness and length ratio in different portions of the antennal gland

The ET consists of LBRs, COEs and the third cell type found in the antennal gland, the end-labyrinthine cell (ELBR) (Fig. 4A). The ELBRs are distributed in the margin of the ET and are often interdigitated with capillaries (Fig. 4B,C; supplementary material Fig. S1). The ultrastructure of the ELBR is substantially different from that of the LBR. The ELBRs have irregular apical microvilli, and mitochondria are distributed throughout the cytoplasmic region. In addition, the numerous basal folding distributed from the basal matrix to the apical region of ELBR. No clear boundary is evident between the sub-apical region and the basal region (Fig. 4D–F).

### The localisation of the ion regulatory proteins in the antennal gland

The antibodies that we used in this study are all heterogeneous antibodies. Western blotting tests showed a single band for each of the four ion regulatory proteins, including Na^+^/K^+^/2Cl^−^ cotransporter (NKCC), NKA, V-type H^+^-ATPase (VHA) and Na^+^/H^+^ exchanger (NHE), with molecular weights of approximately 150, 100, 70 and 70 kDa, respectively (supplementary material Fig. S2).

**Fig. 5.**
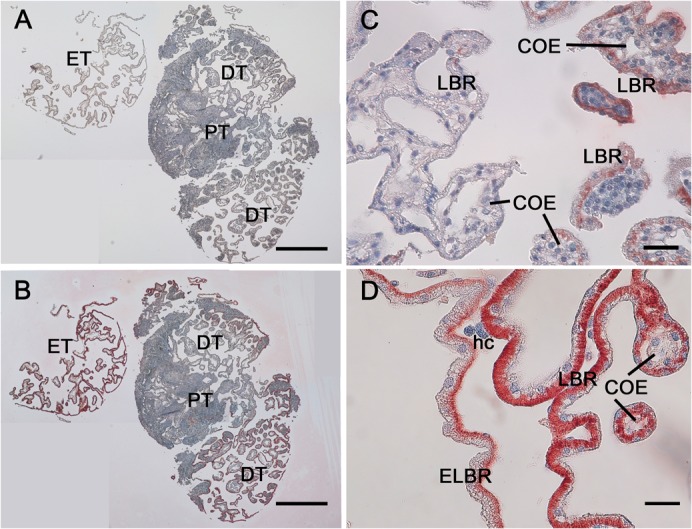
The non uniform distribution of Na^+^, K^+^-ATPase (NKA) in the antennal gland. (A,B) The continuous cross sections of the antennal gland. (A) A negative control slice which was only incubated with the secondary antibody and post stained with the hematoxylin. The blue color indicates the location of the nucleus. (B) The slice showed the localization of NKA in red color which mostly found in the DT and ET regions. (C) The enlarged picture from the PT and DT regions. The labyrinthine cell (LBR) in the PT (left side) shows little staining of NKA than that in the DT (right side). The coelomic cell (COE) shows little staining of NKA. (D) The enlarged picture from the DT and ET regions. The LBR shows a stronger staining pattern than that in the end-labyrinthine cell (ELBR). PT, proximal tubular region. DT, distal tubular region. ET, end tubular region. hc, haemocyte. Scale bars: 250 µm (A,B), 30 µm (C,D).

**Fig. 6.**
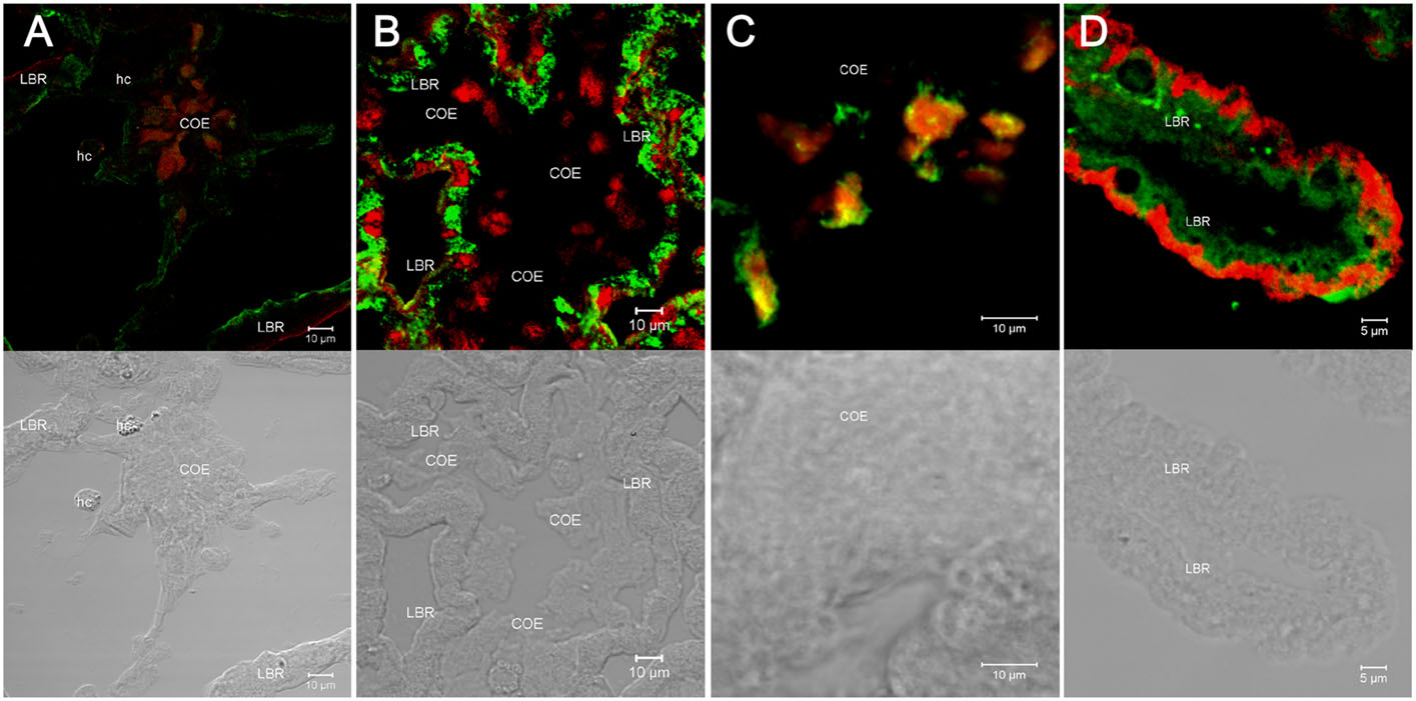
The locations of the ion-regulatory proteins in the proximal tubular region (PT) and the distal tubular region (DT) of the antennal gland. (A) The NKA (green) is in the basolateral membrane and in the foot process (bottom) of the COE in the DT. VHA (red) showed little staining in the apical region of the LBR, with stronger signal aggregated in the centre of the COE. (B) NKA (green) was found in the basolateral membrane of the LBR. NHE (red) was found inside the COE and in the apical to sub-apical regions in the LBR. (C) NKCC (green) and NHE (red) were distributed in the centre of the COE. (D) NKCC (green) was found in the basolateral membrane while VHA (red) was in the apical membrane of the LBR. COE, coelomic cell. LBR, labyrinthine cell. hc, haemocyte. NKCC, Na^+^/K^+^/2Cl^−^ cotransporter. VHA, V-type H^+^-ATPase. NHE, Na^+^/H^+^ exchanger. NKA, Na^+^, K^+^-ATPase. Scale bars: 10 µm (A–C), 5 µm (D).

**Fig. 7.**
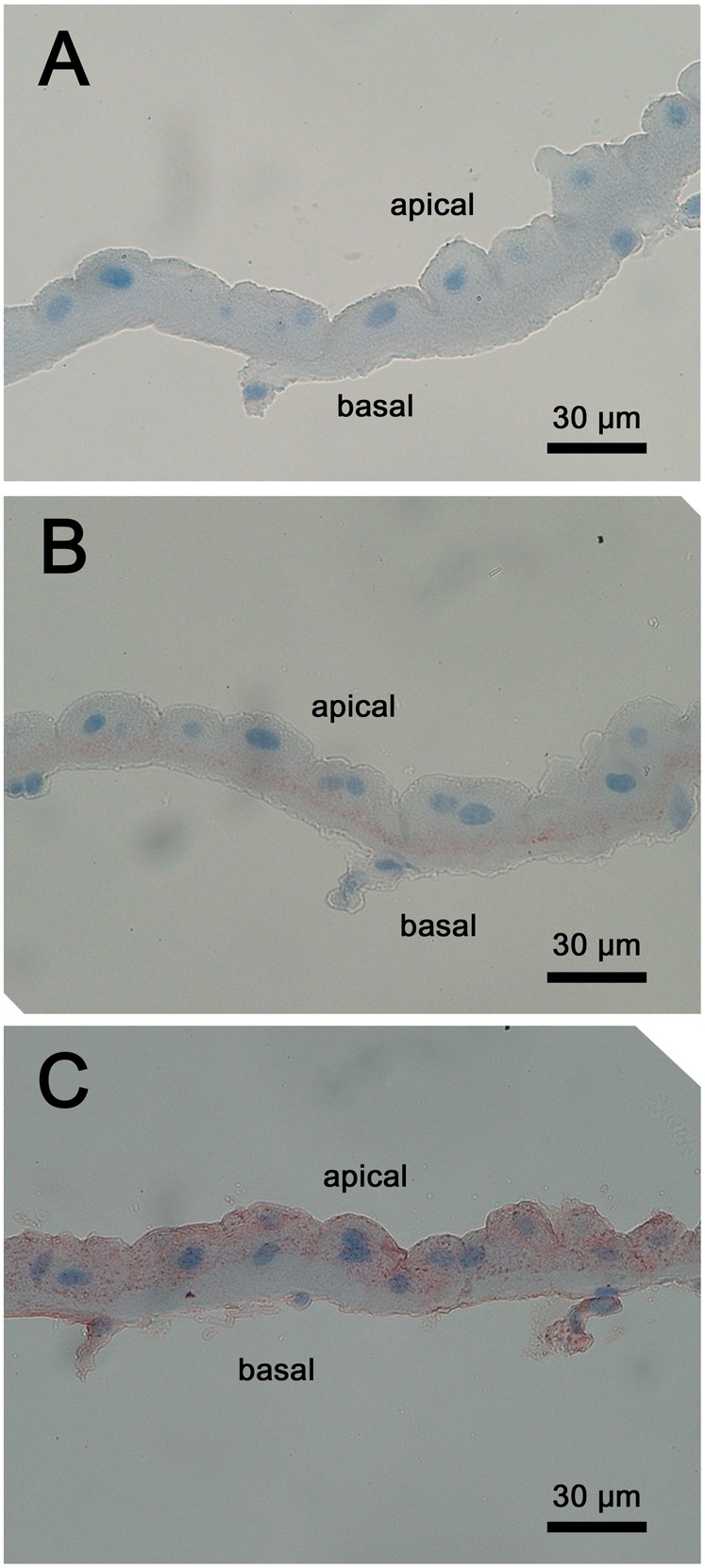
The localizations of Na^+^, K^+^-ATPase (NKA) and Na^+^/K^+^/2Cl^−^ cotransporter (NKCC) in the end-labyrinthine cell (ELBR). The continuous sections (A–C) show different immuno-positive pattern of NKA and NKCC. (A) A negative control slice only incubated with the secondary antibody and post-stained with the hematoxylin. The blue color indicated the location of the nucleus of the ELBR. (B) The red color showed the localization of the NKA in the basolateral membrane (below the nucleus) of the ELBR. The upper side of this image is the apical side, while the bottom is basal side of the cell. (C) The red color indicated the localization of the NKCC-like protein in the apical and sub-apical region (above the nucleus) of the ELBR. The upper side of this image is the apical side, while the bottom is basal side of the cell. Scale bars: 30 µm.

NKA had a non-uniform distribution throughout the antennal gland (Fig. 5), with a higher immunoreactivity in the LBRs and ELBRs of both the DT and the ET than in those of the PT (Fig. 5B–D). VHA and NHE were apically located in both the LBR and ELBR (Fig. 6). However, NKCC was distributed differently in the LBR and the ELBR. It was difficult to distinguish the LBRs from the ELBRs in the cryosections because of the loss of cellular detail. Continuous paraffin sections (2–3 µm) were used to locate the nuclei of the ELBRs and detect NKA and NKCC in the same cells. NKA showed high immunoreactivity in the basolateral membrane of the LBRs but was restricted to the very bottom of the ELBRs (Fig. 5D, Fig. 7B). NKCC was distributed on the apical side of the ELBR (Fig. 7C) but in the basolateral membrane of the LBR (Fig. 6D).

The COEs showed a low NKA immunoreactivity throughout the three regions of the antennal gland (Fig. 5C,D) and were only located in the extended foot process on the bottom of the cell (Fig. 6A). In contrast, NHE, NKCC and VHA aggregated in the centres of the COEs (Fig. 6A–C).

## DISCUSSION

In the past, the gills have been hypothesised to be the principal osmoregulatory organ in brachyuran crabs, and numerous studies have shown that the posterior gills are especially important in ion regulatory processes (Péqueux, 1995; Lucu and Flik, 1999; Chung and Lin, 2006; Henry et al., 2012). However, one of our recent studies has shown that NKA activity in the posterior gills of *O. stimpsoni* did not increase during low-salinity acclimation (Tsai and Lin, 2012), although the activity in the anterior gills did so. In the present study, NKA activity in the antennal gland increased within 4 days after *O. stimpsoni* was transferred to a diluted seawater environment. This result supported the hypothesis that the antennal gland was one of the ion regulatory organs responsible for short-term osmoregulation in the semi-terrestrial crab *O. stimpsoni*.

### Structure–function correlation in the antennal gland

To investigate the ion regulatory mechanism of the antennal gland, it is first necessary to have a good description of the detailed structure of the gland. The lack of studies of the structure and function of the antennal gland in brachyuran crabs hinders comparisons of these characteristics among crustaceans. In a recent review of structure–function relation on the excretory organs, Freire and colleagues summarized the structures among different crustaceans (Freire et al., 2008). For example, the structure of the antennal gland in shrimps and brachyuran crabs is a maze of tubules, while it is a sponge-like tissue in terrestrial amphipods. In crayfish *Procambarus balndingi*, the antennal gland is further divided into the coelomosac (end-sac), labyrinth and nephridial canal (Peterson and Loizzi, 1973; Peterson and Loizzi, 1974; Freire et al., 2008). And, in another crayfish *Pacifasticus leniusculus*, there are a coelomosac, proximal nephron tubule, distal nephron tubule and labyrinth in the antennal gland (Fuller et al., 1989). In the fiddler crab *Uca modax*, the antennal gland is relatively simple compared to those in crayfishes, and only contains a coelomosac and labyrinth (Schmidt-Nielsen et al., 1968).

Several studies have hypothesised that osmoregulatory ability is present in combination with acid–base regulation or organic ion transport in the antennal gland of crustaceans (Wheatly, 1985; Miller et al., 1989; Ahearn and Franco, 1990). However, few of these studies have further elaborated the functional differentiation of the antennal gland. In the brachyuran crab *Metacarcinus magister* (*Cancer magister*), the antennal gland has been found to actively reabsorb Na^+^, K^+^, Ca^2+^, Cl^−^ and HCO_3_^−^ from the urine and to secreted Mg^2+^ during acclimation to 100% seawater. However, when in diluted SW, the antennal gland ceased to secrete Mg^2+^, decreased Ca^2+^ uptake but increases the secretion of H^+^ and HCO_3_^−^ (Wheatly, 1985). In freshwater crayfish, the functions of the segments of the antennal gland are analogous to those of the mammalian nephron (Peterson and Loizzi, 1973; Peterson and Loizzi, 1974). The structures of the cells in the antennal glands of crayfish also provide evidence that active secretion and reabsorption occur (Ueno and Inoue, 1996). Because of the structural complexity of the antennal gland, very few studies are available on the functional differentiation of the cells of the antennal gland in brachyuran crabs.

In the present study, we found a non-uniform distribution of NKA immunoreactivity in the different regions of the antennal gland (Fig. 5), with relatively weak immunoreactivity in the PT and the strongest immunoreactivity in the ET. The non-uniform NKA distribution was similar to that reported in a study of the antennal gland of the crayfish *Astacus leptodactylus* (Khodabandeh et al., 2005b; Khodabandeh et al., 2006), with the proximal tubule showing the lowest NKA immunoreactivity at every developmental stage examined (Khodabandeh et al., 2005a; Khodabandeh et al., 2005c; Buranajitpirom et al., 2010). In the same study, the immunoreactivity of NKA in the COEs of the antennal gland was also found to be low. Although the high level of expression of NKA in the bladder cells was similar to that found in the ELBRs in this study, the cell structure of the ELBRs differed from that of the bladder (Fig. 6).

No distinct borders divide the three regions, and there is no direct evidence that the ELBRs are continuous with the LBRs in *O. stimpsoni*. The apical/basal orientation of the two cell types (LBR and ELBR) is clearly distinct, and they differ in the size of the apical/sub-apical region and the relative location of the nucleus (Table 1; Figs 3, 4). Based on the segmentation and the cell features observed in previous studies, the nephridial canal cells in freshwater crayfish (Fuller et al., 1989) may be analogous to the ELBRs observed in this study. To our knowledge, the nephridial canal can only be found in freshwater crayfish, in which the nephridial canal is separated from the labyrinth (Khodabandeh et al., 2005a). The lack of an intervening tubule in marine-derived crustaceans has also been described (Schmidt-Nielsen et al., 1968; Freire et al., 2008). Not surprisingly, no nephridial canal was found in the semi-terrestrial *O. stimpsoni*. Furthermore, the review of the structure–function relationships of the antennal gland by Freire and colleagues indicates that the apical microvilli in the tubular cells tend to increase in length and density towards the distal end, with increasing basal membrane folding (Freire et al., 2008). In the present study, we also found that the length of the apical microvilli and the total cell thicknesses in the LBR significantly increased from the proximal portion to the distal portion of the antennal gland. However, the ratios (lengths of the apical microvilli/total cell thicknesses) were relatively similar for each cell type (Table 1). This result indicates that the cells increase in size from the PT through the DT to the ET.

### The suggested ion regulatory mechanisms of the cells in the antennal gland

**Fig. 8.**
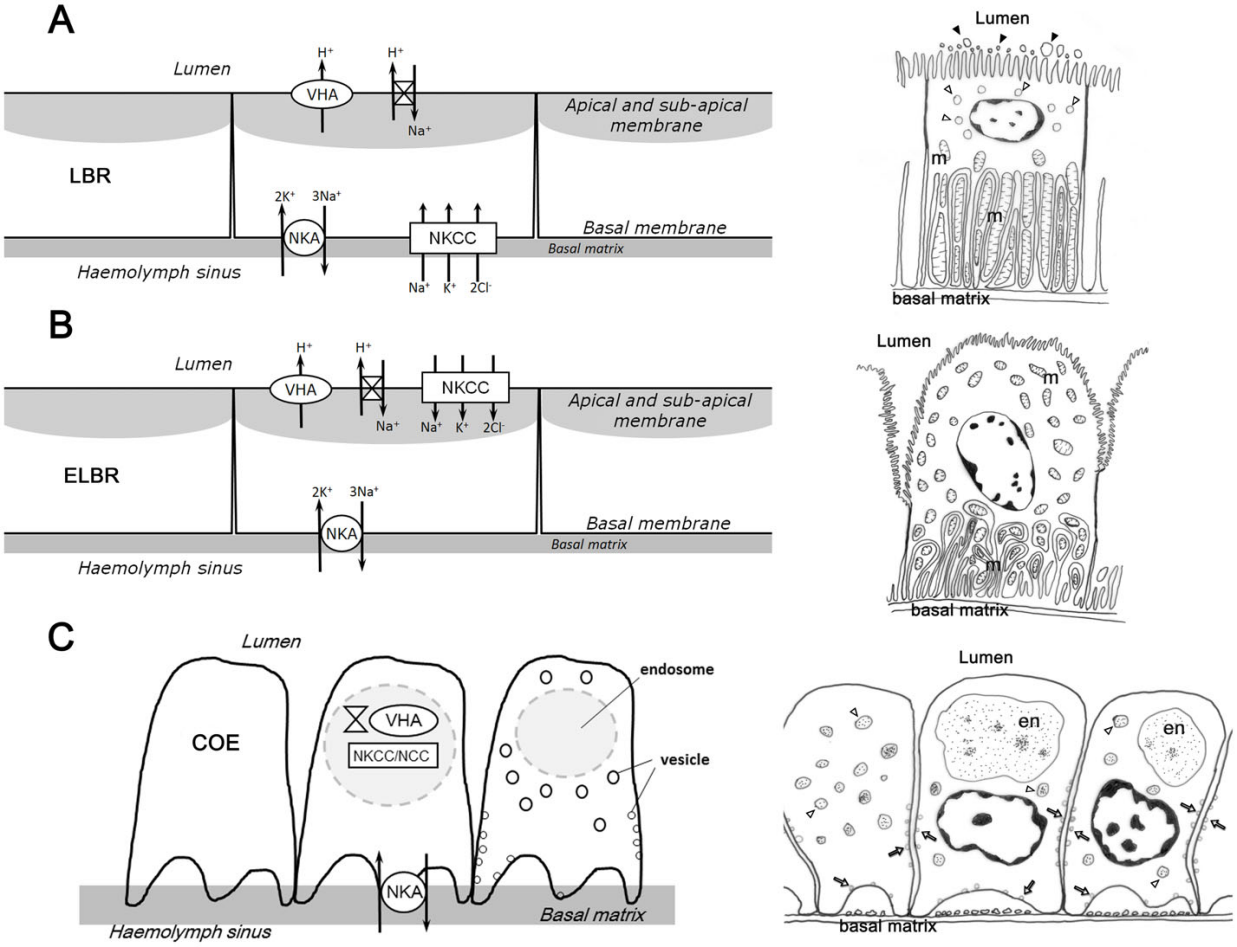
Schematic drawing of the ion-regulatory models and the ultrastructure of the three cell types in the antennal gland. (A) The labyrinthine cells (LBR) have apical brush-border microvilli, a sub-apical space with vesicles (open triangle) and basal folding with elongated mitochondria (m). On the outer side of the apical brush-border microvilli of the LBRs, numerous aposomes (black arrowheads) were frequently found. NKCC and NKA locate in the basolateral membrane. VHA and NHE locate in the apical membrane and in the sub-apical region. (B) The ELBRs have an irregular apical membrane and its mitochondria (m) are distributed in the whole cytoplasm. NKA locates in the basolateral membrane while NKCC in the apical membrane. VHA and NHE are in the apical membrane and in the sub-apical region. (C) The coelomic cells (COE) have small vesicles (open arrow), vesicles (open triangle) and a large endosome (en). NKA locates in the membrane of the foot process. The NKCC, NHE and VHA aggregate in the centre of the COE.

In the two types of cuboidal cells, the LBRs and the ELBRs, NKA was distributed basolaterally, whereas NHE and VHA showed apical localisation (Fig. 8). This type of cellular distribution pattern in the three proteins (NKA, NHE and VHA) can be found in the LBRs and ELBRs of the antennal gland, whereas NKCC shows a different distribution in the LBRs and the ELBRs (Figs 7, 8). This ion regulatory mechanism of the LBRs is similar to what we have found in the epithelial cell of the ion regulatory gill of *O. stimpsoni* (Tsai and Lin, 2012). The ion regulatory models of the LBRs and ELBRs are similar to those for the gills of marine and euryhaline crabs and to that for the posterior gill epithelium of *O. stimpsoni* (Morris, 2001; Tsai and Lin, 2012).

The high immunoreactivity of the antennal gland for Na^+^, K^+^-ATPase (NKA) in crayfish and lobster has suggested its potential role in producing diluted urine (Khodabandeh et al., 2005a; Khodabandeh et al., 2005b). Moreover, the high NKA activity of the antennal gland has been found to be correlated with nitrogenous excretion in *Ocypode quadrata* (De Vries et al., 1994). The apically localised VHA and NHE participate in both the ion regulatory mechanism and the acid–base regulation of aquatic animals (Hwang and Lee, 2007; Freire et al., 2008) as well as the ammonia excretion process (Weihrauch et al., 2009). However, an apically located NKCC (or NCC) is hypothesised to function in ion reabsorption, whereas the basolateral NKCC is hypothesised to function in ion excretion (Freire et al., 2008). Therefore, we hypothesise that the LBRs might play a role in ion excretion and the ELBRs in ion reabsorption. The ion regulatory proteins (VHA, NKCC and NHE) were found to be aggregated at the locations of the endosomal system in the cytoplasm of the COE (Fig. 3E,F, Fig. 6, Fig. 8), suggesting that the ion regulatory proteins may be located in the membrane of the vesicles. More detailed studies are required to clarify the relationship between these transporting proteins and their physiological roles, especially in the COE.

### The possible functional differentiation of the antennal gland

**Fig. 9.**
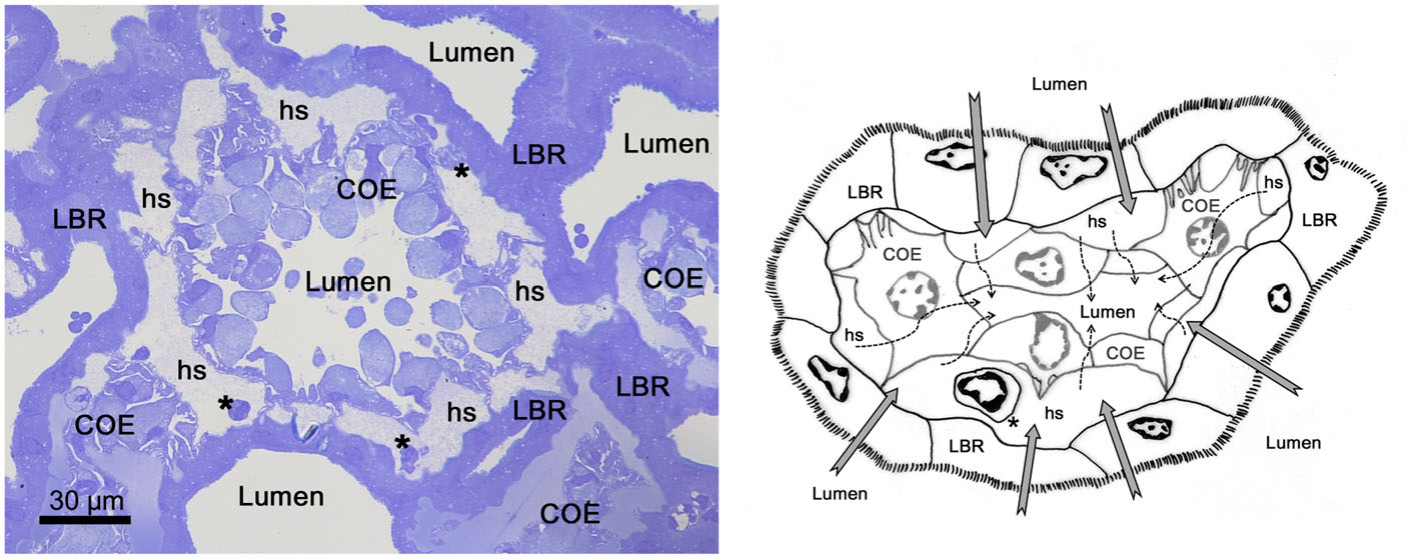
Schematic diagram of the proposed haemolymph/urine flow in the antennal gland. The haemolymph/urine flow first enters into the antennal gland through COEs (dash arrow) into the lumen. The filtrate is transported to the apical side of LBRs and re-processed by the LBRs (gray arrows). COE, coelomic cell. LBR, labyrinthine cell. hs, haemolymph sinus. *haemocyte. Scale bar: 30 µm.

Based on both the morphological and the immunohistological results, it appears increasingly plausible that functional differentiation occurs among the portions/regions of the antennal gland in *O. stimpsoni*. The coelomosac did not exhibit a large end-sac form as found in crayfish (Khodabandeh et al., 2005b). Rather, it branched into the maze of tubules, forming numerous islets in the proximal tubular region (PT). The islet of the coelomosac corresponds to the characteristics found in corrosion cast data on the blue crabs *Callinectes sapidus* and *Cancer magister* (McGaw, 2005). Based on the corrosion casts and our cross-sections of the antennal gland, it is suggested that the haemolymph first enters the antennal gland from the coelomosac artery and ends in the islet-type haemolymph sinus which is surrounded by the basal matrix between the coelomic cells and the labyrinthine cells (Fig. 9). Furthermore, the capillaries (capillary-like vessel) were frequently found and participate in the circulatory system of invertebrates (Goodman and Cavey, 1990; McGaw and Reiber, 2002). We propose the following model of the filtration pathway of the haemolymph and the function of each portion of the antennal gland. In the anterior regions (PT and DT) of the antennal gland, the haemolymph passes into the sinus between the basolateral side of the LBRs and the bottom of the COEs. The filtrates pass through the COEs and are reprocessed by the LBRs via passive ion excretion (Fig. 9). After the filtrates are transported to the posterior region (ET), ion reabsorption occurs, primarily in the ELBRs. The filtrates return to the body circulation via the venous sinus located on the opposite side of the coelomosac artery. We hypothesise that the venous sinus is the end of the capillaries found in the distal tubular region and the end tubular region.

In conclusion, the role of the antennal gland may differ among crabs that live in various habitats. In a semi-terrestrial crab *Ocypode stimpsoni*, the antennal gland is an ion regulatory organ and participates in ion reabsorption from the urine, exhibiting functional differentiation among its segments (PT, DT and ET). The distal and end portions of the antennal gland may play a relatively important role in the ion regulatory process.

## MATERIALS AND METHODS

### Crabs and acclimation

*Ocypode stimpsoni* were collected from Gao-mei wetland in Taichung City, Taiwan (N24°19′25.6″, E120°33′7.9″). All of the crabs were in the intermolt stage with a 30±5 mm carapace width. The molting stage of each crab was judged by both observing the color of the cuticle and the hardness of the carapace. Two to three crabs were kept in plastic container (L74×W50×H25 cm) containing approximately 5 cm depth of 35‰ seawater for at least 1 week before experiment. The total water volume in each treatment is about 18 L. The crabs were free to emerge from the water. The crabs were fed 2–3 times per week with commercial freeze-dried shrimp. The temperature was maintained at 24±3°C with a 14 L:10 D photoperiod and the artificial seawater prepared with Coralife Scientific Grade Marine Salt (OCEANIC, USA). The acclimation medium of different salinities were maintained and changed every two days. Once the necrosis was found in the carapace or in the gills, that specimen will not be used in any experiment. All the experimental animals used in this study were following the guidance of three Rs (Replacement, Reduction and Refinement) and Article 15 of the Animal Protection Law in Taiwan.

### Protein extraction

After seven days in 35 ppt seawater and 1, 4, 7 days in 5 ppt diluted seawater, the crabs were anaesthetised on ice and sacrificed by the destruction of the dorsal ganglia. The antennal glands were cut into small pieces and placed into a homogenization medium (25 mM Tris-HCl, 0.25 mM sucrose, 20 mM EDTA, 0.4% sodium deoxycholate) with a protease inhibitor cocktail (the final concentrations in homogenization medium were: 2 mM antipain, 1 mM leupeptin, 10 mM benzamidine, and 5–10 mM aprotinin (Sigma, USA)) and homogenized using an ultrasonic processor (Sonics, USA). The homogenates were first centrifuged at 4°C and 6,000 *g* for 15 min. The supernatant was then centrifuged again at 4°C and 20,160 *g* for 20 min. The supernatant was to determine NKA activity. A portion of the supernatant was immediately stored at −78°C for western blotting. The assay for protein concentration determination followed the procedure of Tsai and Lin (Tsai and Lin, 2007; Tsai and Lin, 2012) with the BioRad protein assay kit (cat. no. 500-0002, BioRad, USA). The absorbance was read at 695 nm by a spectrophotometer (U-2001, Hitachi, Japan).

### Na^+^, K^+^-ATPase activity

The enzyme-specific activity of Na^+^, K^+^-ATPase (NKA) was defined as the difference between the concentrations of the inorganic phosphates liberated in the reaction medium in the presence and absence of ouabain. The composition of the reaction medium in both experimental groups were 1) ouabain-free group: 20 mM imidazole, 100 mM NaCl, 30 mM KCl, 10 mM MgCl_2_, pH 7.4 and 2) ouabain group: 20 mM imidazole, 130 mM NaCl, 10 mM MgCl_2_, 1 mM ouabain, pH 7.4). The experimental protocol and the calculation of the NKA activity were the same as previous studies (Tsai and Lin, 2007; Tsai and Lin, 2012).

### Immunofluorescent and immunohistological study

After seven days in 35 ppt seawater, crabs were anaesthetised on ice and sacrificed by destroying the dorsal ganglia. The antennal glands were removed and incubated in ice-cold 4% paraformaldehyde (in 0.1 M phosphate buffer) for 4–6 hr at 4°C. For the immunofluorescent experiments, the tissues were washed in phosphate buffered saline three times for 20 min and perfused in 30% sucrose. After the tissues fell to the bottom of the tube, they were embedded in OCT (Tissue-Tek®, CA, USA) for cryosectioning. The sections (5–8 µm) of the antennal gland were washed and incubated with primary antibodies for each ion-regulatory protein. The sections were then incubated with the secondary antibodies that conjugate the fluorescent dye and observed on a fluorescent confocal microscope (Zeiss LSM 510, Germany). For the immunohistological study, the tissues were dehydrated in a graded ethanol and xylene solution and then permuted in liquid paraffin for the subsequent embedding procedure. The sections (3–5 µm) of the antennal gland were incubated with primary antibodies and then stained with a commercial kit containing a 2nd antibody HRP/Fab polymer conjugate and aminoethyl carbazole (AEC) single solution chromogen (PicTure-Plus^TM^, Invitrogen, USA), following the procedure of Tsai and Lin (Tsai and Lin, 2007; Tsai and Lin, 2012). The localization of each protein was examined using a light microscope (E600, Nikon, Japan).

### Histological and electron microscopic study

The crabs were anaesthetised and sacrificed by the same method as in the immunohistological study. The antennal glands for the electron microscopic study were removed carefully and incubated in ice-cold 4% paraformaldehyde with 5% glutaraldehyde (in 0.1 M phosphate buffer) for 4–6 hr at 4°C. The osmolality of the fixative solution was adjusted to 950 mOsm for preventing the cell from shrinking/swelling. After dehydration in a 50%, 75%, 95% ethanol series, tissue samples were treated by 50%, 75% LR White Resin and embedded in 100% LR White Resin (London Resin Company Ltd., England). Semi-thin sections (0.6 µm) were stained with 1% toluidine blue, and the structures of the antennal gland were observed and photographed using a light microscope (E600 Microscope with D1 digital camera, Nikon, Japan) for cell thickness. The ultra-thin sections (90 nm) were stained with 2% uranyl acetate for 15–18 hr, and the ultrastructures were observed and photographed on a transmission electron microscope (Hitachi H-7000, Japan).

### Antibodies

The mouse monoclonal antibody against the α-subunit of the avian sodium pump (α5) and the mouse monoclonal antibody against the human Na-K-Cl cotransporter (T4) were purchased from the Developmental Studies Hybridoma Bank (University of Iowa, Iowa City, IA, USA). The V-type H^+^-ATPase antibody was a rabbit polyclonal antibody raised against a synthetic peptide of the V-type H^+^-ATPase A-subunit. This V-type H^+^-ATPase antibody was kindly provided by Prof. Kaneko (Ocean Research Institute, University of Tokyo, Tokyo, Japan). The Na^+^/H^+^ exchanger antibody was a rabbit polyclonal antibody raised against the dace NHE and was kindly provided by Prof. Hirose (Department of Biological Sciences, Tokyo Institute of Technology, Yokohama, Japan). For the immunofluorescence study, we used a minimal cross-reaction to rabbit proteins Alexa Fluor 488-conjugated AffiniPure F(ab′)_2_ goat anti-mouse IgG antibody (Jackson Immunoresearch, USA) and a Cy3-conjugated goat anti-rabbit IgG antibody (Jackson Immunoresearch, USA) to perform our double labelling experiments. For the immunohistochemical study, we employed a commercial kit containing a secondary antibody HRP/Fab polymer conjugate and an aminoethyl carbazole (AEC) single solution chromogen (Invitrogen, USA).

### Quantitative analysis and statistics

We used Image-Pro Plus (Ver. 4.5, Media Cybernetics, Inc., USA) to determine the thicknesses of the labyrinthine cells and the apical/total length ratio in different portions of the antennal gland. All measurements are expressed as mean values ± S.D. Differences in the cell thicknesses among different regions were analyzed by one-way ANOVA followed by Duncan's multiple range test. SAS software (ver. 9.1) was used.

## Supplementary Material

Supplementary Material
