# Supplementary material for: Functional anatomy and ion regulatory mechanisms of the antennal gland in a semi-terrestrial crab, Ocypode stimpsoni
Source: Biol Open. 2014 May 2;3(6):409–17. doi: 10.1242/bio.20147336 (PMC4058075; doi:10.1242/bio.20147336)
Supplement: Supplementary Material [file supp_3_6_409__index.html]

Functional anatomy and ion regulatory mechanisms of the antennal gland in a semi-terrestrial crab, Ocypode stimpsoni — Functional anatomy and ion regulatory mechanisms of the antennal gland in a semi-terrestrial crab, Ocypode stimpsoni — Supplementary Material 

# Functional anatomy and ion regulatory mechanisms of the antennal gland in a semi-terrestrial crab, *Ocypode stimpsoni*

## bio.20147336 Supplementary Material

**Files in this Data Supplement:**

- Supplementary Material - Jyuan-Ru Tsai and Hui-Chen Lin doi: 10.1242/bio.20147336
