# Supplementary material for: Functional anatomy and ion regulatory mechanisms of the antennal gland in a semi-terrestrial crab, Ocypode stimpsoni
Source: Biol Open. 2014 May 2;3(6):409–17. doi: 10.1242/bio.20147336 (PMC4058075; doi:10.1242/bio.20147336)
Supplement: Supplementary Material [file supp_bio.20147336_bio.20147336-s1.pdf]

Biology Open (2014) 000, 1–9 doi:10.1242/bio.20147336

Supplementary Material

Jyuan-Ru Tsai and Hui-Chen Lin doi: 10.1242/bio.20147336

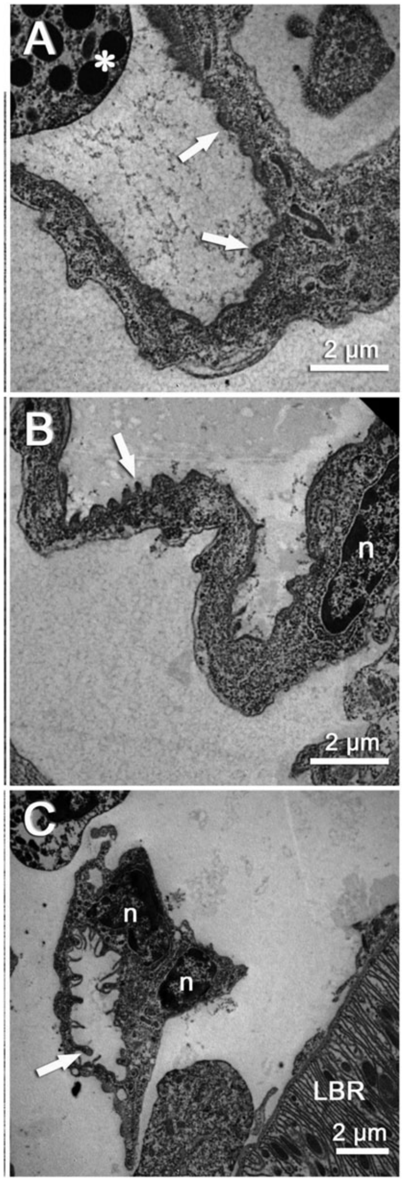

Fig. S1. The ultrastructure of the capillary in the antennal gland. Most of the capillaries are composed of two or more endothelial cells. Haemocytes (*) are found in the capillary and the protuberance (arrows) of the inner side of the endothelial cell is found in large, medium and small (capillaries) (A–C, respectively). n, nucleus. Scale bars: 2 μm.

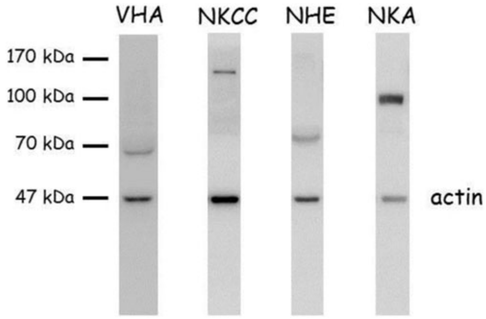

Fig. S2. The western blotting of the ion regulatory proteins in the antennal gland. The single band pattern shows the antigen–antibody specificity in the antennal gland of the *Ocypode stimpsoni*. $Na^+/K^+/2Cl^-$ cotransporter (NKCC) was detected with a molecular weight about 150–160 kDa. Na^+, K^+-ATPase (NKA) was detected with a molecular weight at 100 kDa. Na^+/H^+ exchanger (NHE) was detected with a molecular weight at 70 kDa. V-type H^+-ATPase (VHA) was detected with a molecular weight about 65–70 kDa. The total protein concentration of each lane was 10–20 μg.
